# Interrelationship between the menstrual cycle and the phenomena associated with orthodontic tooth movement in young female patients: a systematic review

**DOI:** 10.1007/s42000-025-00678-8

**Published:** 2025-05-29

**Authors:** Hayat M. R. M. A. Almansour, Athanasios E. Athanasiou, Miltiadis A. Makrygiannakis, Eleftherios G. Kaklamanos

**Affiliations:** 1 Hamdan Bin Mohammed College of Dental Medicine, Mohammed Bin Rashid University of Medicine and Health Sciences, Dubai, United Arab Emirates; 2 School of Dentistry, European University Cyprus, Nicosia, Cyprus; 3 School of Dentistry, National and Kapodistrian University of Athens, Athens, Greece; 4 School of Dentistry, Aristotle University of Thessaloniki, Thessaloniki, Greece

**Keywords:** Orthodontics, Tooth movement, Menstrual cycle

## Abstract

**Purpose:**

Alterations in the levels of female sex hormones throughout the menstrual cycle can affect bone turnover, which, in turn, may influence the phenomena related to orthodontic tooth movement. The aim of the present study was to investigate in a systematic manner and summarize the existing evidence regarding the interrelationship between the menstrual cycle and the phenomena associated with orthodontic treatment in young females.

**Method:**

An unrestricted search was performed. Studies investigating the interdependence between the menstrual cycle in young females and the phenomena associated with tooth movement were reviewed. Study retrieval and selection were followed by extraction of relevant data and risk of bias assessment with the Cochrane ROB 2 tool for randomized controlled studies and the Newcastle-Ottawa Scale for the remaining studies.

**Results:**

Finally, seven studies, with variable risk of bias, met the inclusion criteria. Overall, force application during menstruation led to greater tooth movement. Orthodontic pain increased when forces were applied in the luteal phase and was exacerbated when patients presented with primary dysmenorrhea. Orthodontic treatment did not affect the duration of menstrual bleeding or cycle length in the long term.

**Conclusion:**

The menstrual cycle may exert an effect on the phenomena associated with orthodontic tooth movement: an orthodontist should therefore be able to identify possible repercussions.

**Supplementary Information:**

The online version contains supplementary material available at 10.1007/s42000-025-00678-8.

## Introduction

Adolescent girls and women are reported to constitute the main group of patients who seek orthodontic treatment [1]. Despite their greater eagerness to undergo treatment, it is suggested that females exhibit certain negative differences regarding a number of orthodontic treatment characteristics. For example, in comparison to men, women have been shown to report increased levels of orthodontic pain, an uncomfortable sensation experienced by more than 90% of patients, usually from the third to the seventh day following the application of orthodontic forces [2, 3]. Furthermore, in female patients pain has been reported to last longer [3–5].

Α plausible explanation for this disparity in pain nociception between genders could be attributed to the female gonadal hormones [6, 7]. The menstrual cycle is a normal physiological process that regularly occurs in women, starting from early adolescence, in approximately monthly cycles. Estrogen is one of the most important sex hormones involved in the regulation of the menstrual cycle [8, 9]. The feeling of pain has been shown to fluctuate during menstrual phases [10–12]. In addition, a number of physical and psychosocial factors, such as exercise, nutrition, occupation, and lifestyle, have been linked to menstruation variations [13, 14].

Alveolar bone turnover constitutes the cardinal phenomenon during orthodontic tooth movement [15]. Previous studies have brought attention to the possible alterations in tooth movement due to bone metabolism changes occurring during pregnancy, lactation, ovariectomy, and the estrous cycle in experimental animals [16–18]. It is therefore of importance also to investigate the effects of menstrual cyclical changes on the phenomena associated with orthodontic tooth movement. However, to the authors’ knowledge, no relevant systematic review has to date been conducted. The objective of the present review was to investigate in a systematic way and summarize the existing evidence regarding the interdependence between the menstrual cycle and the phenomena correlated with orthodontic tooth movement in young female patients and to appraise the quality of the pertinent evidence.

## Materials and methods

### Protocol and registration

The protocol of the present systematic review was developed, conducted, and reported according to relevant methodological guidelines [19–22]. It was registered retrospectively on Open Science Framework (https://osf.io/ksrjy/). Ethical approval was not needed since the current investigation is a systematic review.

### Eligibility criteria

The Participant, Intervention, Comparator, and Outcomes (PICOS) domains were used to describe the eligibility criteria. Studies included were prospective (randomized or not) controlled studies investigating the interdependence between the menstrual cycle (e.g., specific stage of the cycle, duration of menstrual bleeding, menstrual cycle length, and dysmenorrhea) and phenomena associated with orthodontic tooth movement (e.g., rate of tooth movement, pain, and biomolecule levels). The included studies needed to have recruited healthy young females (with menstrual cycle) undergoing orthodontic tooth movement at various stages of the cycle as their sample. Animal studies, ex vivo, in vitro, in silico, non-comparative research (case reports and case series), and reviews (traditional reviews, systematic reviews as well as meta-analysis) were excluded from the present investigation (Supplementary Table 1).

### Information sources and search strategy

The following electronic databases were searched: MEDLINE via PubMed, CENTRAL, Cochrane Systematic Reviews, Scopus, Web of Science^™^ Core Collection and ProQuest Dissertations & Theses Global databases. For each database, one of the authors devised specific search strategies. They were all based on the MEDLINE strategy but were modified to take into consideration differences in controlled vocabulary and syntax rules for each database (Supplementary Table 2). No restrictions were placed on language, date, or status of publication. In addition, efforts were made to obtain conference proceedings and abstracts, where possible, and the reference lists of all the eligible studies for additional records were searched.

### Selection process

The titles and abstracts of the located records, followed by the full report of any record considered to meet the inclusion criteria, were assessed independently and in duplicate by HMRMAA and MAM, who were not blinded. If the abstract was unclear, the full paper was accessed to determine eligibility for inclusion.

### Data collection and data items

Two of the authors extracted data separately and any disagreements were addressed through discussion with another author. To record the desired information, they used data collection forms which included the studies’ bibliographic information, details about the study designs and verification of study eligibility, participant characteristics (number, age, etc., where available), tooth movement model, details on outcome characteristics and results, and additional information, such as a priori sample size calculation. If clarifications or additional material were required regarding the published data, attempts would be made to contact the corresponding authors.

### Study risk of bias assessment

- The risk of bias in the included randomized controlled trials was assessed independently and in duplicate by the first and fourth authors, with the RoB2 tool [23]. The risk-of-bias VISualization (robvis) web application was then used to enter the assessments [24]. For the non-randomized studies, the risk of bias was assessed using the Newcastle-Ottawa Quality assessment scale for Cohort Studies [25]. Disagreements were resolved through discussion in all the processes mentioned above; kappa statistics were not calculated because of relevant suggestions [22].

### Effect measures, synthesis methods, certainty assessment, and additional analyses

Although a synthesis of the results was to be conducted, it finally was not carried out on account of methodological diversity. Due to inadequate information, analyses for “small-study effects”, publication bias, and subgroup analyses were also not performed [22].

## Results

### Study selection

**Fig. 1:**
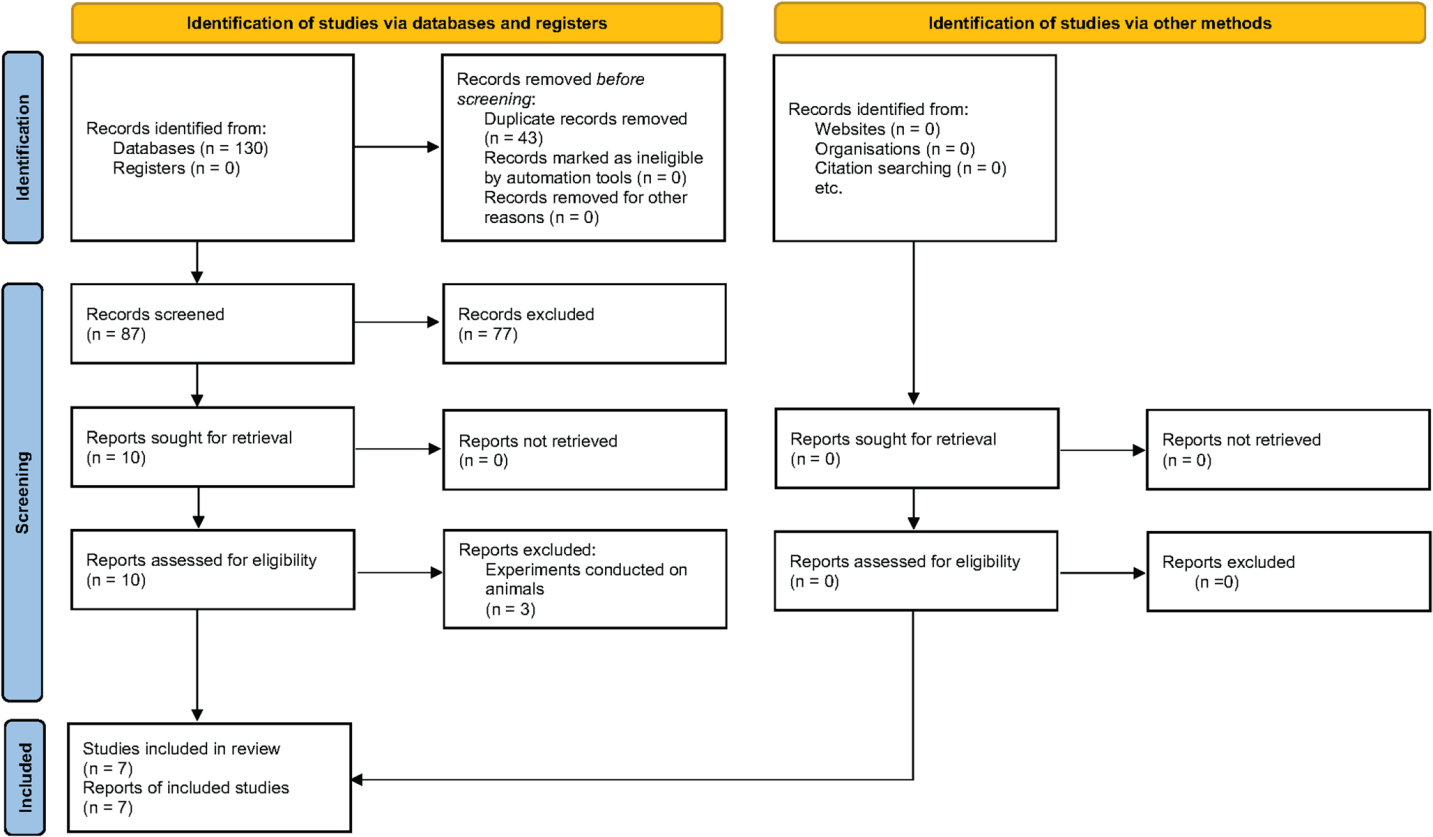
Flow of records

Figure 1 shows the flowchart of records as they go through the review process. The data search took place in December 2024. Initially, 130 records were identified through the database search. Of these, 43 were excluded as duplicates and 10 were considered for inclusion. After elimination of three studies conducted in animal subjects (Supplementary Table 3), seven records were finally included in the systematic review [26–32].

### Study characteristics

Tables 1 and 2 present the features of the retrieved studies. The studies were published between 2014 and 2024 and included two randomized [30, 31] and five cohort studies [26–29, 32].

**Table 1:** General characteristics of the studies included in the systematic review

Study	Intervention characteristics	Outcomes & assessment methods	Power calculation
**Duan et al. 2016** Cohort study	Initial engagement of a 0.012” NiTi archwire *Orthodontic treatment group* *No treatment group*	**Menstrual cycle characteristics** Menstrual cycle length, duration of menstrual bleeding, and amount of blood lost	Yes
**Ileri et al. 2016** Cohort study	Extractions of Mx 4s and 3s distal movement [lacebacks] *G1: The menstrual cycle’s follicular phase* *G2: the menstrual cycle’s luteal phase*	**Orthodontic pain** VAS; days 1, 2 following initial activation **Verbal rating scale**	Yes
**Long et al. 2017** Cohort study	Initial engagement of a 0.012” NiTi archwire *G1: The menstrual cycle’s follicular phase* *G2: the menstrual cycle’s luteal phase*	**Orthodontic pain** VAS; days 1, 2, and 3 following initial engagement	Yes
**Peruga & Lis 2024** Cohort study	SS Transpalatal arch between Mx 6s *G1: menstrual phase (day 3 of cycle)* *G2: ovulation phase day (day of ovulation)* *G3: luteal phase (7 days after ovulation)*	**Intermolar width** Measured between mesiobuccal cusps of molars **Molar rotation**	Yes
**Wang et al. 2014** RCT [split mouth]	Extractions of Mx 4s and 3s distal movement [0.018 × 0.025 SS; 150 g; NiTi spring attached on mini screws] *G1: menstrual phase activation side* *G2: ovulation phase activation side*	**Rate of tooth movement** Measured on casts; distance of the canine apex and the mesiobuccal cusp of the maxillary first molar on the same side after 4 weeks of force application	NM
**Yang et al. 2014** RCT	Extractions of Mx 4s and 3s distal movement [0.018 × 0.025 SS; 150 g; NiTi spring attached on mini screws] *G1: menstrual phase activation group* *G2: ovulation phase activation group*	**E2**,** OCN**,** RANKL OPG levels in GCF** Sampling on the distal side of the canine on day 0 (T0), as well as 15 (T1), 30 (T2), and 45 days (T3) later	NM
**Ye et al. 2014** Cohort study	Initial engagement of a 0.014” NiTi archwire *3 groups according to primary dysmenorrhea*	**Orthodontic pain** VAS; days 1, 2, 4, 7, 14, and 28 following initial engagement	NM

E2: Estrogen; G: group; GCF: gingival cervical fluid; NiTi: nickel-titanium; NM: not mentioned; OCN: osteocalcin; OPG: osteoprotegerin; RANKL: receptor activator of nuclear factor- κB ligand; RCT: randomized controlled study; SS: stainless steel; VAS visual analog scale; 3(s): canine(s); 4(s): first premolar(s); 6(s); first molar(s)

**Table 2:** Sample characteristics in the studies included in the systematic review

Study	Inclusion and exclusion criteria	Analyzed sample
**Duan et al. 2016** Cohort study	**Inclusion criteria**: MCL & DMB fluctuates no more than 3 days and 1 day respectively for at least 1 year, no dysmenorrhea or other reproductive system disease, no oral diseases other than teeth irregularity **Exclusion criteria**: Smokers or alcoholics, suffering from psychological diseases, suffering from systematic diseases, using medications, requires orthognathic surgery and using contraceptives	**164 [**18-40y]
**Ileri et al. 2016** Cohort study	**Inclusion criteria**: American Society of Anesthesiologists (ASA) physical status, regular menstrual cycles, scheduled to undergo the extraction of two upper first premolars **Exclusion criteria**: History of psychiatric treatment, mild-to-severe periodontal disease, a history of orthodontic treatment, difficulty in communication, irregular menstrual cycles, amenorrhea, pregnancy, history of combined oral contraceptive use, pain in any part of the body on the appointment day, and concomitant use of an analgesic within the previous 24 h	**48** [16-20y]
**Long et al. 2017** Cohort study	**Inclusion criteria**: Good general health, regular menstrual periods **Exclusion criteria**: Previous orthodontic Tx, recent orofacial pain, pregnancy, recent medical history, uses contraceptives	**76** [> 18y]
**Peruga & Lis 2024** Cohort study	**Inclusion criteria**: Never been pregnant before, regular menstruation every 28–30 days, Angle Class II with mesial rotation of teeth 16 and 26, absence of acute carious lesions and chronic inflammation in the oral cavity **Exclusion criteria**: Nicotinism, past orthodontic treatment with fixed appliances, missing teeth, hypodontia, and reduced periodontal bone level	**120** [20-30y] BMI [18.5–24.9]
**Wang et al. 2014** RCT [split mouth]	**Inclusion criteria.** Have menarche and regular menstrual cycle, Class I, requiring maxillary first premolar extraction **Exclusion Criteria**: Severe dentition crowding, canine relationship was of significant Class II and III or crossbite relationship	**12** [14-18y]
**Yang et al. 2014** RCT	**Inclusion criteria**: Age 18 to 28 years old, good physical condition, not pregnant, no systemic diseases, no use of hormones or immunomodulators in the past 6 m, regular physiological cycles in the past 12 m, good oral hygiene/periodontal conditions, no history of orthodontic treatment	**12** [18-28y]
**Ye et al. 2014** Cohort study	**Inclusion criteria**: Mild crowding < 4 mm in each jaw, no extraction, no other malocclusions, no crowding, no pregnancy or abortion, regular menstrual cycle, with painful menstrual cycles experienced **Exclusion criteria**: Had previous orthodontic Tx, had analgesic 3 days prior orthodontic Tx, recent toothache, excessive anxiety according to the T-AI ≥57, abnormal CPT < 3 s or > 60 s and abnormal pain tolerance > 5 min	**124** [> 18y]

BMI; body mass index; DMB: duration of menstrual bleeding; CPT: cold pressor test; m: months; MCL: menstrual cycle length; RCT: randomized controlled study; Tx: treatment; T-AI: Trait-Anxiety Inventory; y: years

The two randomized controlled trials involved individuals with maxillary premolar extractions. Wang et al. (2014), in a split-mouth study design, investigated the effects of orthodontic force applied at different stages of the menstrual cycle in adolescent females (14 to 18 years old) in terms of tooth movement rate [30]. After extraction of the first maxillary premolars, the left and right maxillary canines of the same patients were randomly grouped into the menstrual period activation group and the ovulation activation group. All 12 patients were being treated with the MBT™ brackets and underwent alignment until a 0.018 × 0.025” stainless steel wire could be fully engaged. In the menstrual phase activation side, a distal force of 150 g was applied on the canine with a NiTi spring, attached on mini screws, on the first day of the menstrual period and for a period of 4 weeks. In the ovulation phase activation side, the same was performed on the first day of ovulation (as measured by an ovulation test strip). Alginate impressions were taken on the day of force application and 4 weeks later. The distance of the canine apex and the mesiobuccal cusp of the maxillary first molar on the same side was measured. Each measurement was taken six times and was then averaged.

Yang and coworkers (2014) studied the levels of estrogen (E2), osteocalcin (OCN), receptor activator of nuclear factor-κ B ligand (RANKL), and osteoprotegerin (OPG) in the gingival crevicular fluid (GCF) in adult females (18 to 28 years old) [31]. All patients were treated with MBT™ brackets and underwent alignment until a 0.018 × 0.025” stainless steel wire could be fully inserted. Following randomization, six patients were assigned to the menstrual activation group and six patients to the ovulation activation group. In the menstrual activation group, a 150 g distally oriented force was exerted on the right canine with a NiTi spring, attached on mini screws, on the first day of the menstrual period. In the ovulation activation group, a similar force was applied on the first day of ovulation (measured by ovulation test strip). Periodontal scaling was carried out in all patients 1 week before force application and oral health advice was given. The gingival crevicular fluid from the distal side of the right canine was sampled before force application on day 0, as well as on days 15, 30, and 45. Each sampling was repeated three times and averaged.

Peruga and Lis (2024) included three groups of young women in different phases of their menstrual cycle (menstruation, ovulation, and luteal phase) [29]. All included patients had a class II malocclusion and a TPA was placed between the maxillary first molars. The authors measured changes in molar positioning after 6 weeks.

Three studies [27, 28, 32] focused on the overall theme of pain experience after the exertion of orthodontic forces. Long and co-workers (2013) investigated the effect of applying orthodontic force during the follicular or the luteal phase of the menstrual cycle following initial archwire placement [28]. A total of 76 consecutive adult female patients were recruited and were assigned to a follicular or a luteal phase intervention group according to their menstrual cycle stage on the day of bonding. Orthodontic pain levels were evaluated by a visual analog scale (VAS) on days 1, 2, and 3 following initial engagement of a 0.012” NiTi archwire.

Similarly, Ileri et al. (2016) investigated the effect of applying orthodontic force during the follicular or the luteal phase of the menstrual cycle on canine distalization with lacebacks [27]. A total of 48 consecutive adult female patients were recruited and, depending on their menstrual cycle stage on the day of bonding, they were assigned to a follicular or a luteal phase intervention group. Orthodontic pain levels were assessed by a visual analog scale (VAS) on days 1 and 2 following initial activation.

Ye and coworkers (2014) aimed to examine whether a relationship exists between primary dysmenorrhea (PD) and pain experience by the application of orthodontic forces [32]. Orthodontic treatment was initiated within 1 week following the end of the previous menstrual period. Orthodontic pain levels were assessed by a visual analog scale (VAS) on days 1, 2, 4, 7, 14, and 28 following initial engagement of a 0.014” NiTi archwire.

Finally, Duan and coworkers (2016) investigated the effect of bonding brackets and engaging a 0.012” NiTi archwire on menstrual cycle length, duration of menstrual bleeding, and amount of blood lost in adult female patients [26]. Data were collected over six consecutive cycles from a group of 164 women with normal cycles.

### Risk of bias within studies

**Fig. 2:**
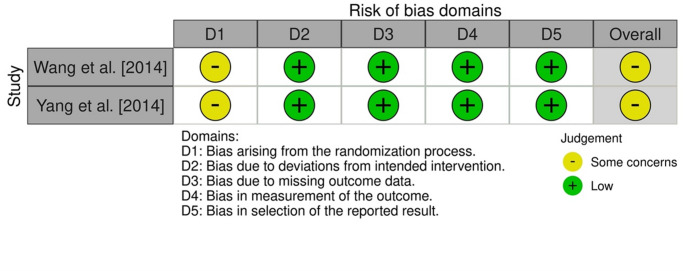
Risk of bias assessment, according to ROB 2 tool

The risk of bias assessment for the two randomized studies showed some concerns regarding the randomization process (Fig. 2), resulting in similar rating for the overall risk of bias. The summary of risk of bias assessment for the non-randomized studies is presented in Table 3. All five included studies received the maximum score.

**Table 3:** Summary of risk of bias assessment for the non-randomized studies

Quality assessment criteria	Duan et al. [2016]	Ileri et al. [2016]	Long et al. [2017]	Peruga & Lis [2024]	Ye et al. [2014]
Representativeness of exposed cohort?	*	*	*	*	*
Selection of the non-exposed cohort?	*	*	*	*	*
Ascertainment of exposure?	*	*	*	*	*
Demonstration that outcome of interest was not present at start?	*	*	*	*	*
Study controls for age/sex?	*	*	*	*	*
Study controls for at least 3 additional risk factors?	*	*	*	*	*
Assessment of outcome?	*	*	*	*	*
Was follow-up long enough for outcome to occur?	*	*	*	*	*
Adequacy of follow-up of cohorts?	*	*	*	*	*

### Results of individual studies

Regarding the effects of orthodontic force applied at different stages of the menstrual cycle, it was shown that teeth move faster when the orthodontic forces are applied during the menstrual period, when estrogen levels are low [30]. Similarly, Peruga and Lis (2024) observed that intermolar width increased more in the group in which the TPA was activated during the menstrual phase [29]. Yang et al. (2014) showed that estrogen and OCN levels in the ovulation activation group were significantly higher than those in the menstrual activation group (*p* < 0.05), promoting faster tooth movement [31]. No differences were found in RANKL and OPG levelsor in the RANKL/OPG ratio (*p* > 0.05).

Pain was reported to be greater when forces were applied during the luteal phase than during the follicular phase of the menstrual cycle [27, 28]. Ye and coworkers (2014) reported that there was a positive correlation between the severity of primary dysmenorrhea and the intensity of orthodontic pain [32]. Females who experienced more menstrual pain tended to experience orthodontic pain with greater intensity and for a longer period.

Finally, Duan and co-workers (2016) observed that the length of the menstrual cycle may be affected by fixed orthodontic treatment in the first month after bonding, but there was no effect during follow-ups [26]. When compared to baseline, the length of the first menstrual cycle was considerably extended, by 2.1 $\:\pm\:$*p* = 0.003). No differences were shown in duration or amount of bleeding.

### Additional analyses

Subgroup analyses, as well as analysis of “small-study effects” and publication bias, were not possible due to lack of data.

## Discussion

### Summary of available evidence

**Fig. 3:**
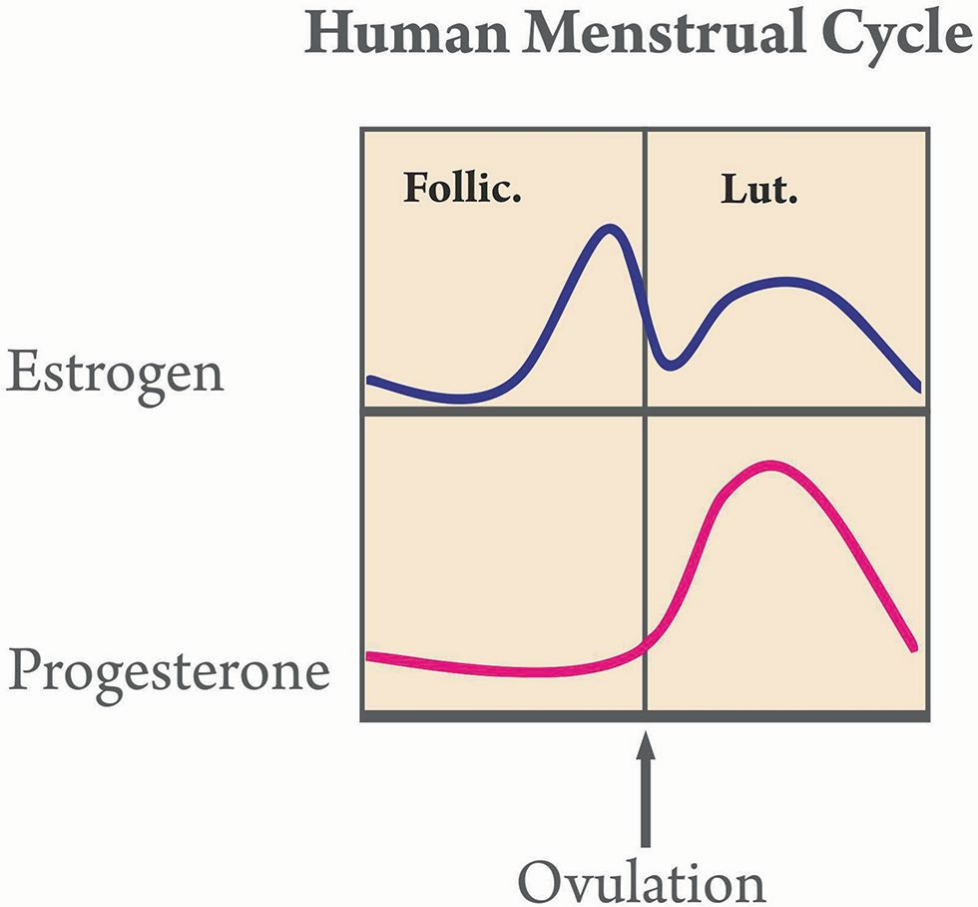
Hormone fluctuation during the human menstrual cycle

Fluctuation of hormone levels, as those encountered during the menstrual cycle, may affect alveolar bone metabolism [33]. Overall, tooth movement increases during the menstrual part of the cycle, in which estrogen and/or progesterone levels are lower (Fig. 3). Pain levels associated with orthodontic force application have been reported to be lower during the same period. Females who feel very severe menstrual pain tend to experience orthodontic pain with greater intensity and for longer time periods. Finally, menstrual cycle characteristics (length of cycle, duration, or amount of bleeding) were not affected long-term by the exertion of orthodontic forces. Based on the above-mentioned observations, orthodontists should take into account the possibility that female patients may respond differently during the various stages of the menstrual cyclewhile also considering the potential repercussions.

When orthodontic forces are applied during the menstrual period, teeth move faster [30]. At this stage, estrogen and progesterone levels are lower. Estrogens are bone resorption inhibitors and promote bone mass maintenance [34]. In fact, estrogen plays a critical role in bone structure modification, bone mass maintenance, and the bone tissue remodeling process [35]. Previous research has shown that there is a link between the speed of tooth movement and osteoclastic activity [36], which can be inhibited by estrogen [37]. Furthermore, the reorganization of periodontal tissue during tooth movement involves the reconstruction of periodontal fibers, whose deposition and cross-linking of the collagen fibers can be affected by estrogen [38]. Apart from estrogen, progesterone has also been associated with bone preservation, either directly through its action on osteoblasts or indirectly through its impact on glucocorticoid receptors and metalloproteinases [39]. Progesterone has been associated with a slower pace of tooth movement [40].

Estrogen levels naturally fluctuate during a woman’s menstrual cycle. They are lowest during menstruation and peak around ovulation while following a periodic monthly pattern. When estrogen levels are high (during ovulation), osteoblast function prevails and osteoclast function is low, resulting in a greater rate of osteogenesis compared to bone resorption, which could lead to a deceleration of orthodontic tooth movement. In contrast, when estrogen levels are low (during the menstrual phase of the cycle), osteoclast function dominates and osteoblast function is low, resulting in a rate of bone loss which is greater than that in osteogenesis, enabling a higher rate of orthodontic tooth movement. Estrogen levels after menopause are low; therefore, women in this age group are more susceptible to osteoporosis symptoms [41]. As a result, knowing how the menstrual cycle affects tooth movement in orthodontics and evaluating the implications could well enhance the work of the orthodontist.

A previous systematic review on animal studies concluded that tooth movement was increased in the stages of the estrus cycle in which the estrogen and/or progesterone levels were lower [18]. The rate of movement was reported to be higher in animals in estrus when estrogen and progesterone levels are said to be at their nadir. In contrast, the tooth movement was lower in proestrus animals when estradiol levels peak [42, 43]. Moreover, negative correlations were observed between estradiol and serum TRAP activity, as well as pyridinoline, both of which are markers of bone resorption [42]. The fluctuating pattern of serum progesterone differed from that of estradiol, with the peak occurring in diestrus. Its lowest levels were measured during estrus, similarly to estradiol [42]. Serum osteocalcin, a bone development marker, demonstrated substantial correlation with progesterone [42].

Studies investigating the overall issue of pain associated with orthodontic treatmentshowed that pain levels are higher in patients in the luteal phase than in those in the follicular phase of the cycle when estrogen and progesterone levels are at their nadir [27, 28]. The effects of female sex hormones on pain have previously been investigated, the results, however, being inconsistent [6, 7, 44]. Ring et al. (2009) and Ahmed et al. (2012) have reported that pain is less intense when estrogen levels are lower [10, 45]. Arab and co-workers (2015) and de Kruijf and co-workers (2016) reported contrasting results [11, 46], although the observed discrepancies might be the result of other variables in pain perception, apart from female gonadal hormones. Anxiety and quality of life have been shown to alter orthodontic patients’ perception of discomfort [30, 47]. Moreover, education levels have been found to be negatively associated with patients’ first-day orthodontic pain levels, indicating that patients with higher educational levels may perceive less pain [48].

Women with severe primary dysmenorrhea tend to experience persistent pain sensation following the application of orthodontic forces, suggesting that dysmenorrhea may potentially serve as a preliminarily predictor of persistent pain [32]. Orthodontic pain and dysmenorrhea share common biomolecular pathways. Calcitonin gene-related peptide, which increases the feeling of pain by triggering the release of substance P and the induction of orthodontic pain [49–51], is highly expressed in patients with dysmenorrhea [52].

Regarding the duration of the menstrual cycle, Duan et al. (2016) demonstrated that the initiation of a fixed orthodontic therapy may lead to elongation of the first menstrual cycle during treatment but does not affect the subsequent cycles [26]. Other characteristics such as the duration or amount of bleeding were not shown to be affected. The influence of fixed orthodontic treatment on menstrual cycle length is not yet clear, although previous studies have revealed a negative effect of stress and psychological factors on the menstrual cycle [53].

Although the data set is limited, it is evident that it would be useful for the orthodontist to consider the possibility that menstruating patients could exhibit differential physiological bone remodeling during different phases of the menstrual cycle, this having potential clinical implications. In terms of mechanotherapy, it is important to remember that patients may require more anchorage preparation if force is applied during those phases when estrogen levels are at their lowest. In contrast, activation during these stages may promote tooth movement, thereby shortening the total duration of orthodontic treatment. While it is hypothesized that fixed appliances should be removed when high amounts of estrogen or progesterone are circulating, this was not explicitly studied in the material collected. Finally, it is clear that the phase of the cycle significantly affects how females perceive orthodontic pain and that the severity of menstrual pain can be a marker of the pain associated with orthodontic forces.

### Strengths and limitations of the present review

The adoption of a well-established technique is one of the review’s strengths. The data retrieval approach was comprehensive, with no pre-set constraints on language, publication date, or status. To avoid potential biases, screening, eligibility verification, information abstraction, and bias risk assessment were all conducted twice, and any disagreements were resolved through conversation until a final agreement was reached. The limitations stem mostly from the characteristics of the studies included in it and the data gathered during the review process. Furthermore, the use of specific treatment modalities to induce orthodontic tooth movement limits the applicability of the acquired data to other clinical situations.

### Recommendations for future research

As female patients constitute the majority of orthodontic patients, it is evident that more well-designed experimental studies to examine and gain insight into the effect of the menstrual cycle on orthodontic tooth movement would be of considerable use to clinicians. Standardization of study designs is highly desirable [54]. Furthermore, future research should simulate, as closely as possible, scenarios in clinical practice in humans in terms of force magnitude as well as regarding the characteristics of the force delivery method used.

## Conclusions

The menstrual cycle and orthodontic tooth movement appear to influence each other. Specifically, teeth tend to move faster and osteocalcin (OCN) levels are higher when orthodontic forces are applied during menstruation, a phase characterized by low estrogen levels. Additionally, pain related to orthodontic treatment may be more intense during the luteal phase (after ovulation) than during the follicular phase (before ovulation). Women who typically experience stronger menstrual cramps might also feel orthodontic pain more severely and for longer durations. Furthermore, initiating fixed orthodontic treatment may temporarily affect the length of the first menstrual cycle after the appliances are bonded, though this effect does not persist in later cycles. Given these interactions, orthodontists should be mindful of the menstrual cycle phase when planning treatment, as it could impact patient comfort and treatment effectiveness.

## Electronic supplementary material

Below is the link to the electronic supplementary material.

Supplementary Material 1
