# Supplementary material for: Interrelationship between the menstrual cycle and the phenomena associated with orthodontic tooth movement in young female patients: a systematic review
Source: Hormones (Athens). 2025 May 29;24(4):1183–92. doi: 10.1007/s42000-025-00678-8 (PMC12678451; doi:10.1007/s42000-025-00678-8)
Supplement: Supplementary file 1 — Supplementary Material 1 [file 42000_2025_678_MOESM1_ESM.docx]

**Supplementary Table 1.** Inclusion and exclusion criteria

| **Domain** | **Inclusion criteria** | **Exclusion criteria** |
| --- | --- | --- |
| **Participants** | - Healthy female orthodontic patients (with menstrual cycle) |  |
| **Interventions** | - Any kind of orthodontic intervention with fixed appliances |  |
| **Comparisons** | - Comparisons between menstrual cycle phases - No orthodontic intervention | - Lack of control group |
| **Outcomes** | - Quantitative data regarding characteristics of the menstrual cycle and the phenomena associated with orthodontic tooth movement (e.g., rate of tooth movement; pain; intensity of pain; duration of phases of menstrual cycle etc.) | - No relevant quantitative data |
| **Study design** | - Randomized clinical trials, cohort and case-control studies. | - Animal studies, ex vivo, in vitro, in silico, non-comparative research - Systematic reviews and meta-analyses |

**Supplementary Table 2.** Search strategies for each database.

| **Database [2024 12 05]** | **Search strategy** | **Hits** |
| --- | --- | --- |
| **General Sources** |  |  |
| **PubMed** | (orthodon* OR “orthodontic force” OR “mechanical force”) AND (menses OR menstrual OR menstruat*) | **33** |
| **Cochrane Central Register of Controlled Trials** | (orthodon* OR "orthodontic force" OR "mechanical force") AND (menses OR menstrual OR menstruat*) in Title Abstract Keyword | **3** |
| **Cochrane Database of Systematic Reviews** | (orthodon* OR "orthodontic force" OR "mechanical force") AND (menses OR menstrual OR menstruat*) in Title Abstract Keyword | **0** |
| **Scopus** | TITLE-ABS-KEY((orthodon* OR "orthodontic force" OR "mechanical force") AND (menses OR menstrual OR menstruat*)) | **42** |
| **Web of Science™** | (orthodon* OR “orthodontic force” OR “mechanical force”) AND (menses OR menstrual OR menstruat*) (Topic) and Preprint Citation Index (Exclude – Database) Timespan: All years. Search language=Auto | **29** |
| **Grey literature sources** |  |  |
| **ProQuest Dissertations and Theses Global** | (orthodon* OR “orthodontic force” OR “mechanical force”) AND (menses OR menstrual OR menstruat*) Limit: Orthodontics [Full text] | **23** |

**Supplementary Table 3.** Studies excluded after checking full-text.

| **Excluded [after full-text assessment]** | **Reason of exclusion** |
| --- | --- |
| Haruyama N, Igarashi K, Saeki S, Otsuka-Isoya M, Shinoda H, Mitani H. Estrous-cycle-dependent variation in orthodontic tooth movement. J Dent Res. 2002;81:406–10. | Animal study |
| Tan Z, Zhao Q, Chen Y. The mutual effects between orthodontic tooth movement and estrous cycle or estrogen. Biol Rhythm Res. 2010;41:75–81. | Animal study |
| Celebi AA, Demirer S, Catalbas B, Arikan S. Effect of ovarian activity on orthodontic tooth movement and gingival crevicular fluid levels of interleukin-1β and prostaglandin E(2) in cats. Angle Orthod. 2013;83:70–5. | Animal study |

**Supplementary Table 4.** All studies assessed for eligibility.

| **Title** | **Year** | **Journal** | **Authors** |
| --- | --- | --- | --- |
| Development and Validation Method of Analysis of Etoricoxib and Paracetamol in Effectiveness Combination in Tablet Dosage Form | 2021 |  | Al Mufty, Maha and Abu Dayyih, Wael |
| Çocuklarda sürmekte Olan Birinci büyük azı dişlerinin dişeti oluğu sıvısının Proteomik Analizi | 2023 |  | Albat, Selin and Akyüz, Serap |
| Force Control in Bodily Movement of the Lower First Molar in the Monkey Macaca mulatta | 1983 |  | Alexander, C. Moody |
| Does the rate of orthodontic tooth movement change during the estrus cycle? A systematic review based on animal studies | 2021 | BMC Oral Health | Almidfa, N. S. S. and Athanasiou, A. E. and Makrygiannakis, M. A. and Kaklamanos, E. G. |
| Biomechanical Applications of a Novel Simple Machine | 2024 |  | Andreucci, Carlos Aurelio and Fonseca, Elza Maria Morais and Jorge, Renato Manuel Natal |
| Relatório de Estágio Profissionalizante Farmácia Ferreira de Sousa | 2015 |  | Brilhante, Michael Quinta and Fernandes, Lucinda |
| Ethnic Differences in the Upper Lip Response to Incisor Retraction | 2000 |  | Brock, Ralph A. |
| Assessing Temporomandibular Joint Hypermobility in a Young Population: A Pilot Study | 2023 |  | Burgstahler, Elizabeth Rose and Beyer, John |
| The role of endothelin-2 in ovulation | 2015 |  | Cacioppo, Joseph A. and Ko, CheMyong J. |
| Movimento Ortodôntico: Efeito dos Analgésicos | 2012 |  | Carvalho, Sérgio Filipe Lopes and Queirós, Maria Gabriel |
| Effect of ovarian activity on orthodontic tooth movement and gingival crevicular fluid levels of interleukin-1β and prostaglandin E<sub>2</sub> in cats | 2013 | ANGLE ORTHODONTIST | Celebi, Ahmet Arif and Demirer, Serhat and Catalbas, Bulent and Arikan, Sevket |
| To Evaluate the Clinical and Esthetic Outcome of Human Chorionic Membrane Allograft with Coronally Advanced Flap in the Management of Isolated Gingival Recession | 2018 |  | Choudhury, Suhail Ahmed and K.J, Nisha |
| Planeamento de um estudo comparativo da qualidade de vida em pacientes com aparatologia ortodôntica fixa versus alinhadores invisíveis na consulta de ortodontia do Instituto Universitário Egas Moniz | 2020 |  | Costa, Catarina Maria Ramos da and Costa, Hélder Nunes |
| Análise Comparativa Entre Tratamento Ortodôntico com Alinhadores vs Tratamento Ortodôntico Convencional: Revisão Narrativa | 2023 |  | de Gioanni, Martina and Gião, Ana |
| The Influence of le Fort 1 Maxillary Advancement Osteotomy on Nasal Morphology and Aesthetics | 2021 |  | de Sequeira Fernandes, Natascha Lília Margarida and dos Santos Pereira, Rui Manuel and e Silva Jardim, Luís Filipe de Almeida |
| The Effects of Sex-Steroids and Menstrual Cycle/Oestrous Phases on Knee Ligament Laxity in Humans and Rodents | 2014 |  | Dehghan, Firouzeh and Salleh, Naguib |
| Influence of fixed orthodontic treatment on the menstrual cycle of adult females: <i>A prospective longitudinal study</i> | 2016 | ANGLE ORTHODONTIST | Duan, Peipei and Qu, Wenwen and Zou, Shujuan and Chen, Yangxi and Lan, Hui and Farella, Mauro and Mei, Li |
| Relationship between body mass and dental and skeletal development in children and adolescents | 2013 |  | DuPlessis, Elizabeth A. and Araujo, Eustaquio A. |
| Antibioterapia Profilática na Extração de Terceiros Molares Inclusos | 2016 |  | Fernandes, José António de Abreu Monteiro Martins |
| Efeitos da fluoxetina sobre a odontogénese e o desenvolvimento dos tecidos mineralizados dos dentes | 2020 |  | Fonsêca, Déborah Daniella Diniz and Monteiro, Carlos |
| Measuring Regional Changes in Damaged Tendon | 2011 |  | Frisch, Catherine Kayt Vincent and Vanderby, Ray, Jr. |
| Relatório de Atividade Clínica | 2016 |  | Garcia, Marta Inês Quintans and Baptista, André and Veiga, Nélio |
| Effect of reducing the incremental distance of tooth movement per aligner while maintaining overall rate of movement on self-reported discomfort in Invisalign patients | 2012 |  | Garrett, Justin Alexander and Buschang, Peter H. |
| Relevância da Periodontologia na Ortodontia | 2011 |  | Gaspar, Inês de Carvalho and Pinho, Mónica Morado |
| Developing a socio-dental system of dental needs assessment in children | 2004 |  | Gherunpong, Sudaduang |
| Development of topical thalidomide for aphthous ulcers of HIV /AIDS patients | 2003 |  | Gordon, Sharon Michelle and Flexner, Charles W. |
| Physiopathology of osteoclast in bone | 2008 |  | Gu, Xiaomei and Everett, Eric T. |
| [An experimental study on the relationship between the orthodontic tooth movements and menstrual cycle] | 2007 | Shanghai Kou Qiang Yi Xue | Guo, J. and Che, X. X. and Zhao, Q. and Chen, Y. X. |
| Effects of the rhythm of estrogen during estrous cycle on periodontal tissue remolding induced by orthodontic tooth movement大鼠动情周期不同阶段正畸牙移动牙周改建调控机理的比较研究 | 2005 |  | Guo, Jie Z. |
| Effects of female related hormones on orthodontic tooth movement | 2023 | Chinese Journal of Orthodontics | Han, W. and Guo, L. and Li, S. |
| Estrous-cycle-dependent variation in orthodontic tooth movement | 2002 | JOURNAL OF DENTAL RESEARCH | Haruyama, N. and Igarashi, K. and Saeki, S. and Otsuka-Isoya, M. and Shinoda, H. and Mitani, H. |
| Quality and intensity of pain associated with continuously applied orthodontic stresses of relatively high and low magnitudes | 2011 |  | Hentscher-Johnson, Jodi K. and Iwasaki, Laura |
| The Effect of Digital Diagnostic Setups on Orthodontic Treatment Planning | 2017 |  | Hou, Derek J. and Huang, Greg J. |
| Evaluation of Salivary RANKL in Menopausal Women With Periodontitis: A Cross Sectional Study | 2016 |  | K. B, Smitha and S, Ravindra |
| Age Estimation of Adolescents and Young Adults by Radiographic Assessment of Mandibular Third Molar | 2005 |  | Kapoor, Madhur R. |
| Orthodontic Anchorage: Exploration Using a Finite Element Model | 1992 |  | Kellam, Stephen A. |
| Evaluating Trends in the Timing of Cervical Vertebral Maturation in Caucasian Girls | 2020 |  | Kim, Jamie and Leggitt, Leroy |
| Avaliação da Eficácia da Micro-Osteoperfuração na Retração Canina: Revisão Sistemática | 2023 |  | Kouhous, Houssam and Urzal, Vanda |
| Study of the Variations of Eruption of Deciduous Teeth in Children | 2010 |  | Kundu, Surajit |
| Association Between Body Mass Index and Permanent Tooth Development in Hispanic Children in Houston, Texas | 2020 |  | Lam, Andrea and Pazmiño, Katherine |
| The effects of menstrual phase on orthodontic pain following initial archwire engagement | 2017 | ORAL DISEASES | Long, H. and Gao, M. and Zhu, Y. and Liu, H. and Zhou, Y. and Liao, L. and Lai, W. |
| In Vivo Identification of Periodontium MSCs and Their Response to Periodontitis | 2019 |  | Luo, Wenjing and Zhao, Hu |
| DEVELOPMENT AND CHRONIC DISEASE: FUNCTIONAL ADAPTATION IN CYSTIC FIBROSIS (MATURATION, GROWTH, AUXOLOGY) | 1984 |  | Mahaney, Michael Charles |
| The effect of microcurrent stimulation on ATP synthesis in the human masseter as evidenced by phosphorus-31 magnetic resonance spectroscopy | 2005 |  | Mannheimer, Jeffrey S. and Zipp, Genevieve Pinto |
| Transcutaneous Electrical Nerve Stimulation Therapy in Temporomandibular Disorder – A Clinical Study | 2006 |  | Moger, Ganapathi L. and Shashikanth, M. C. |
| Pharmacological Interventions for Pain Relief During Orthodontic Treatment | 2016 |  | Monk, Aoife |
| Psychophysiological reactivity to stress in nail biters | 1998 |  | Morley, Debra Sue and Mostofsky, David I. |
| Mammary gland involution as a target for pregnancy-associated breast cancer prevention: Insights from NSAIDs and macrophages | 2010 |  | O'Brien, Jenean H. and Schedin, Pepper J. |
| A secular increase in the tempos of tooth formation: 1980–2010 | 2012 |  | O'Neill, Kevan Michael and Harris, Edward F. |
| Treatment Outcomes Relative to Predetermined Treatment Objectives | 1998 |  | Opin, Gary Louis and Nanda, Ravindra |
| Apical Root Resorption during Orthodontic Therapy | 1964 |  | Orr, Robert Lee |
| Comparação da qualidade de vida do paciente, reabsorção radicular, higiene oral e impacto no periodonto utilizando alinhadores vs. Aparelho fixo convencional – revisão bibliográfica | 2020 |  | Pereira, Carolina Serra and Costa, Teresa Sobral |
| Biologia da Remodelação óssea | 2010 |  | Pereira, Victor and Figueiredo, Maria Helena Lopes |
| Are Currently Selected Laboratory Animals Useful in the Research of How Female Hormones Influence Orthodontic Biomechanics? | 2023 | Animals (Basel) | Peruga, M. and Kawala, B. and Sarul, M. and Kotowicz, J. and Lis, J. |
| Correlation of sex hormone levels with orthodontic tooth movement in the maxilla: a prospective cohort study | 2024 | Eur J Orthod | Peruga, M. and Lis, J. |
| Influência da Prática de Natação em Alta Competição na Situação Periodontal | 2011 |  | Quintal, Joanna Andreia Rodrigues |
| Evaluation of Modified Microosteoperforation on the Rate of En Masse Retraction. A Prospective Clinical Study | 2018 |  | Rasid, Shaeeb and M.B, Halkati |
| Descriptive Exploratory Study of Individuals’ Use of Pulsed Electromagnetic Fields, the Micro-Pulse, for Pain Relief | 2019 |  | Ravid, Stacey and Barry, Charlotte |
| Estudo da Associação Entre a Doença dos Pequenos Vasos Cerebrais e a Doença Periodontal | 2023 |  | Ribeiro, Tiago Saturnino Amaral Pinto and de Faria e Almeida, Ricardo Manuel Casaleiro Lobo and da Silva Junqueira Polónia, Jorge Manuel and de Campos Felino, António Cabral |
| The multiple impulse method of tooth mobility assessment | 1995 |  | Robertson, R. G. |
| Dental Anxiety in Future and Current Orthodontic Patients | 2011 |  | Roy, Joanie and Dempster, Laura |
| Effect of Transverse Dimensional Changes on the Health of the Buccal Bone in the Maxillary Arch of Using Passive Self-Ligating Brackets Using CBCT: An in Vivo Study | 2019 |  | S, Vandana and C.S, Ramachandra |
| Development of Functional Spheres for Bone Regeneration | 2020 |  | Santos, Carlos Alexandre Barros and Lopes, Maria Ascensão Ferreira Silva |
| Tratamento Ortodôntico em Pacientes com Doença Periodontal | 2016 |  | Santos, Joel Machado and Pinho, Mónica Morado |
| Circulatory MicroRNA-27, -146, and -214 in Gingival Crevicular Fluid During Orthodontic Tooth Movement | 2020 |  | Seagraves, Amy Lynn and Atsawasuwan, Phimon |
| The Influence of Childhood Obesity on Oral Inflammation | 2021 |  | Silver, Chad M. and Nicholas, Christina |
| Vitamin D and chronic pain: A comprehensive review | 2013 |  | Singer, Jonathan A. and Jefferies, Steven |
| A dor nos adolescentes em tratamento ortodôntico com recurso a aparatologia fixa: relação com a motivação e expectativa | 2015 |  | Sousa, Inês Querido de and Costa, Hélder Nunes |
| Deciduous Tooth Emergence, Maternal and Infant Condition, and Infant Feeding Practices in the Brazilian Amazon | 2017 |  | Spence, Jennifer Emily and Piperata, Barbara and Guatelli-Steinberg, Debbie |
| The mutual effects between orthodontic tooth movement and estrous cycle or estrogen | 2010 | Biol Rhythm Res | Tan, Zhen, Zhao, Qing and Chen, Yangxi |
| Comparison of a behavioral intervention with and without a cognitive component in the treatment of body -focused repetitive behaviors | 2003 |  | Teng, Ellen J. and Woods, Douglas W. |
| The Measurement of Fetal Craniofacial Growth by Computer Graphic Centroid Morphanalysis | 1983 |  | Trenouth, M. J. |
| Alt yarı sürmüş 3.molar diş çekimi sonrası trombositten zengin fibrin uygulamasının alveolit insidansı ve cep derinliği üzerine etkilerinin değerlendirilmesi | 2014 |  | Ünsal, Hamiyet and Sayan, Nejat Bora |
| Complicações Cirurgicas do Terceiro Molar | 2017 |  | Vulcano, Elvira and de Macedo, José |
| Üç-Boyutlu Biyobasım Için Yeni Kompozit Biyomürekkep Geliştirilmesi ve Karakterizasyonu | 2021 |  | Vurat, Murat Taner and Elçin, Yaşar Murat |
| Orthodontic tooth movement at different stages of adolescent female menstrual cycle | 2014 | Chinese journal of tissue engineering research | Wang, B. and Yang, X. and Zhou, J. P. and Feng, G. and Dai, H. W. and Huang, L. |
| The Contemporary Coben Analysis | 2010 |  | Wertz, Robert G. and Tuncay, Orhan C. and Viechnicki, William |
| Mediation of the Oral Microbiome Between Obesity and Oral Health in Chicago Children | 2023 |  | Whyms, Pamela S. and Williams, Sloan |
| The effects of surgery on facial growth in bilateral cleft lip and palate Sri Lankan subjects | 2004 |  | Worrell, Emma Caroline |
| Fractal analysis on dental radiographs to detect trabecular patterns in patients affected by periodontitis | 2007 |  | Xiang, Sophia Sy-Hann and Nowzari, Hessam |
| A new approach to accelerate orthodontic tooth movement in women: Orthodontic force application after ovulation | 2010 | Med Hypotheses | Xu, X. and Zhao, Q. and Yang, S. and Fu, G. and Chen, Y. |
| [Preliminary study on the best-exerted force chance in the female menstrual cycle] | 2014 | Hua Xi Kou Qiang Yi Xue Za Zhi | Yang, X. and Dai, H. and Wang, B. and Huang, L. |
| Primary dysmenorrhea is potentially predictive for initial orthodontic pain in female patients | 2014 | ANGLE ORTHODONTIST | Ye, Rui and Wang, Sheng and Li, Yu and Wu, Ruixian and Pei, Jiao and Wang, Jue and Zhao, Zhihe |
| Evaluation of Preemptive Valdecoxib Therapy on Discomfort Caused by Initial Archwire Placement in Adult Orthodontic Patients | 2002 |  | Young, Andrew N. |
| Skausmo, Sukelto Ortodontinio Gydymo, Malšinimo Būdai | 2018 |  | Zapalskytė, Greta and Lopatienė, Kristina |
| Cortical bone thickness of black and white American adolescents | 2012 |  | Zhang, Ningning and Buschang, Peter H. |
| Фармаколошка Анализа Ефеката Селективних Инхибитора Поновног Преузимања Серотонина На Мотилитет Хуманог Јајовода | 2019 |  | Милосављевић, Милош H. and Фолић, Марко |
